# Photo‐Controlled Dynamics of Cholesteric Polymer Coatings via Hydrazone Crosslinking

**DOI:** 10.1002/anie.202507358

**Published:** 2025-06-05

**Authors:** Alexander Ryabchun, Yunita Florida, Quan Li, Rémi Plamont, Nathalie Katsonis, Ivan Aprahamian

**Affiliations:** ^1^ Stratingh Institute for Chemistry University of Groningen Nijenborgh 3 Groningen 9747AG The Netherlands; ^2^ Department of Chemistry 6128 Burke Laboratory Dartmouth College Hanover New Hampshire 03755 USA

**Keywords:** Adaptive materials, Chiral polymer networks, Helix, Molecular switches, Structural colors

## Abstract

Developing responsive coatings and materials requires discovering a breadth of mechanisms by which external stimuli can be converted into useful signals. Here, we demonstrate an approach driven by supramolecular mechanochemistry, where mechanical input—molecular shape change—is translated into structural color variation. By embedding bistable, negatively photochromic hydrazone photoswitches into cholesteric polymer networks, we achieve a reversible, stable color shift through molecular‐scale pulling and pushing of the photonic scaffold. Unlike azobenzene‐based systems, which typically disrupt liquid crystal order, this approach modifies the pitch of a cross‐linked cholesteric helix without disrupting the organisation of the material. The long‐lived stability of both hydrazone isomers ensures durable optical switching. This effect provides a new strategy for designing mechanoresponsive photonic coatings and tunable optical materials.

## Graphical abstract

Here, we present an innovative approach, which integrates responsive molecules into larger ensembles, resulting in photo‐controllable and photo‐patternable colors. By covalently coupling hydrazone photoswitches with supramolecular helical polymer networks, we harness light‐driven molecular shape changes to modulate structural color. Our multilength scale design enables macroscopic level function and potential applications in tunable photonics and optics.

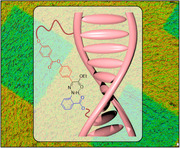

The understanding and subsequent use of the mechanisms of dynamic transduction of information from molecular to micro‐ and macroscopic length scales in novel intelligent materials has garnered much attention in the past decade.^[^ ^1^, ^2^ ^]^ The main motivation behind this research focus is the need to push modern materials science to the next level by designing artificial materials that not only possess a number of passive useful properties (e.g., color, elastic modulus, viscosity, etc.) but can also perform more sophisticated tasks, such as responding to external stimuli, adapting to environmental conditions and even performing computational tasks. In pursuing this goal, scientists have been taking inspiration from natural (biological) materials, where the operation of small molecules and molecular motors govern the properties and functions of bulk materials through a complex network of hierarchical supramolecular organization. As a man‐made counterpart of these biological active molecule's one can consider artificial molecular switches and rotary motors as the triggers that can ultimately be used in mimicking biological functions.^[^ ^3^, ^4^, ^5^, ^6^, ^7^ ^]^ Such molecules can undergo considerable structural changes, and in a reversible manner, when interacting with appropriate stimuli, which can in general be either light or chemical reactants. Despite major advances in the synthesis of molecular motors and switches, a central challenge remains: developing principles that translate changes in molecular structure into functional responses at larger length scales. For example, controlling shape transformation and mechanical properties are crucial for applications such as actuators and soft robotics.^[^ ^8^, ^9^, ^10^ ^]^

Here, we report the integration of molecular switches into optical coatings, where the functional response is driven by the operation of a negatively photochromic hydrazone.^[^ ^11^, ^12^ ^]^ The geometrical photoswitching of the hydrazone is transformed into dynamic color change of the polymer coating by means of multiscale hierarchical design, where the photoswitch is covalently incorporated into the periodic supramolecular helical architecture of a liquid crystal (LC).^[^ ^13^ ^]^ The LC is responsible for the structural coloration of the coating, and a similar principle of coloration can be found in nature (e.g., the color of butterfly and bug wings, feathers, chameleon skin, etc.) and can be described by the following equation (Equation 1):

(1) $$\lambda_{\max}=\mathrm{nP}$$

where *λ*_max_ is the center of reflection band, *n* is the average refractive index, and *P* is the supramolecular pitch. As evident from Equation (1) the reflected color (*λ*_max_) is fully governed by the periodicity of the cholesteric helix (P) which, in turn, is determined by the chirality of the molecules (dopants) inducing a cholesteric mesophase. Molecular photoswitches and rotary motors have been successfully used in controlling P and consequently colors of a material though the modulation of the helical twisting power (HTP; HTP = 1/P*c, where c is concentration of chiral dopant).^[^ ^14^, ^15^ ^]^ Despite the efficiency of this approach, it is only applicable for liquid materials since it requires the rearrangement of supramolecular helices through the modulation of the structure of the chiral dopants.^[^ ^16^, ^17^, ^18^ ^]^ On the other hand, in cross‐linked polymeric systems, the helical rearrangements are hindered by covalent networks and account for a set of mechanical and photophysical properties, which allows their use as coatings, for example. For cross‐linked cholesteric systems, we suggest considering Equation (1) in a different way:

(2) $$\lambda_{\max}=\mathrm{nh}/N$$

where h is the thickness of the layer/coating, and N is the number of full turns of a cholesteric helix, i.e., P = h/N. It is clear then that the covalent network imposes certain restrictions, namely N is constant and cannot be changed. Consequently, the modulation of thickness (h) provides a way to control the color of the layer/coating. Herein, we show how hydrazone photoswitches can be used in varying the thickness of helical polymer layers (Figure 1a) and subsequently tuning their colors.

**Figure 1:**
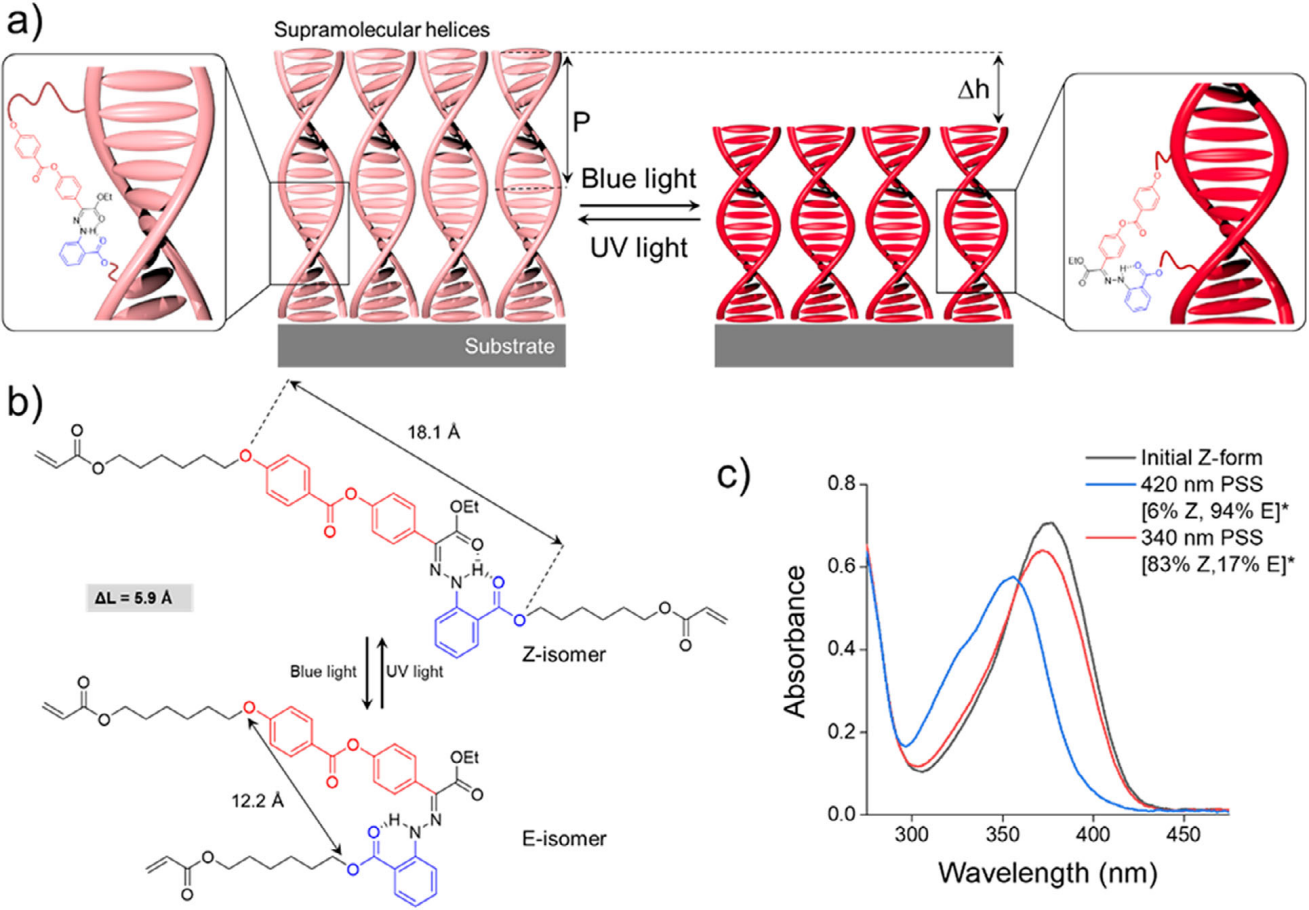
a) Hydrazone photoswitches integrated in a cholesteric liquid crystal polymer network. The change of shape the of the hydrazone drives the contraction/expansion of the network and associated change of pitch in the helix. b) Reversible photo‐induced *Z*/*E*‐isomerization of the artificial molecular switch. The photoisomerization is accompanied by large geometrical changes of the molecular switch, with a difference in molecular length ≈0.6 nm.^[^ ^19^ ^]^ c) Absorbance spectra of the *E* and *Z* isomers of the hydrazone obtained photochemically by exposure to blue (*λ* = 420 nm) and UV light (*λ* = 340 nm), respectively. * Exact *Z*/*E* ratios were obtained by ^1^H NMR spectroscopy analysis.

We used a previously designed hydrazone diacrylate^[^ ^19^ ^]^ for shape encoding and actuating the polymer films (Figure 1b). This hydrazone is negatively photochromic with a thermodynamically stable *Z*‐form. Initially, the yellowish *Z*‐hydrazone isomerizes to the colorless *E*‐form (Figure 1c, see details in Supporting Information, Figures S1 and S2) upon blue light exposure (420 nm). Back isomerization can be accelerated by UV illumination (340 nm), while thermal relaxation under dark is substantially hindered, as the half‐life of the *E*‐form is thousands of years.^[^ ^20^, ^21^ ^]^ The ratios of *Z* and *E* forms obtained in the photostationary state (PSS) are shown Figure 1c.

The hydrazone diacrylate (3 wt.%) was integrated into the cholesteric network obtained by the photocrosslinking of a cholesteric monomeric mixture consisting of mesogenic diacrylate (C6M, 5 wt.%), low molar mass liquid crystals (ZLI1083, 62 wt.%), chiral dopant (CB15, 30 wt.%), and traces of the photoinitiator Irgacure 819 (Figure 2a). The details of the cholesteric coating preparation as well as the data regarding how the composition of the coating affects its optical performance are discussed in the Supporting Information (Figure S3).

**Figure 2:**
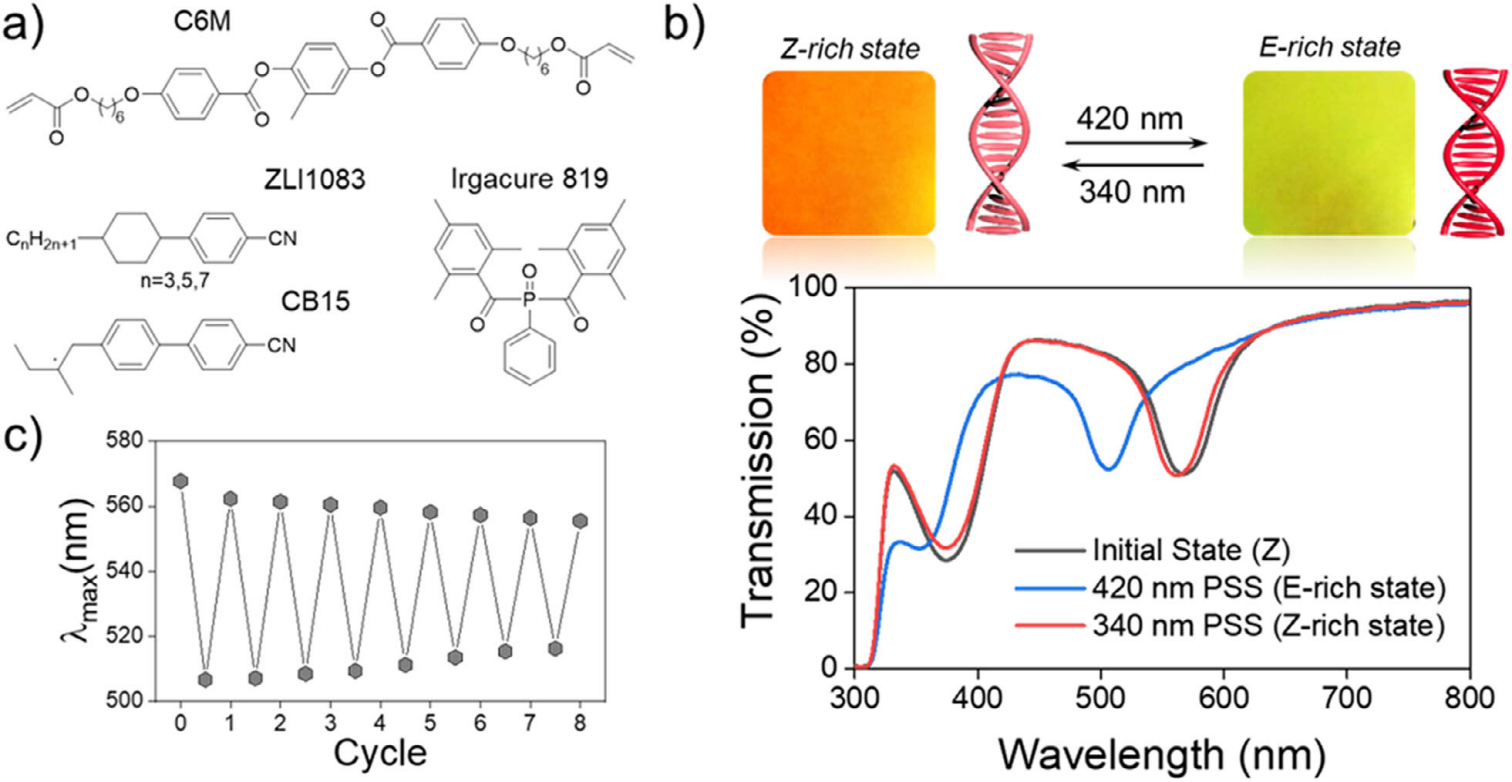
a) Components of the cholesteric liquid crystal polymer network. b) Reflection colors of the cholesteric network containing hydrazone cross‐linker upon light exposure (on top) and corresponding transmission spectra (bottom). The size of the images on top is 1 cm^2^. c) Reversibility of the color change (position of selective light reflection peak, *λ*_max_) during cycling illumination between blue (420 nm) and UV (340 nm) light. All measurements were performed after irradiation, with the light source switched off, ensuring that the observed effects result purely from photochemical Z–E isomerization and not from photothermal heating.

The prepared cholesteric coating is orange colored because of the selective light reflection from the periodic helical structure. The light reflection appears as a band in the transmission spectrum centered at about 570 nm (Figure 2b). Irradiation of this coating with blue light (420 nm) causes a fast color shift, and the coating turns yellow (reflection λ_max_ ∼ 505 nm). Further, irradiation with UV light (340 nm) shifts the band to almost the initial state, restoring the initial color of the coating. The kinetic curves of the color modulations are shown in Figure S4 (see Supporting Information). It is worth noting that if the cholesteric layer is confined between two glass substrates, the reflection band loses its sensativity towards light illumination (Figure S5). However, if one side of the layers is free and unconstricted, the network can contract/expand without any constrains and consequently change color.

The reversible color shift (Δ*λ*_max_) of 65 nm solely originates from the large molecular shape change of the hydrazone (Figure 1b) caused by *Z*–*E* photoisomerization. Because of the geometrical constrains of the glass substrate (i.e., the layer cannot contract/expand in the plane of the substrate), this molecular shape change pulls on the network, thus contracting it in the direction of the coating normal, *i.e*., modulation of layer thickness. Since the number of turns of the cholesteric helix is fixed by the network upon photopolymerization, the decrease in thickness of the layer results in the shortening of the cholesteric pitch and consequently the color of the layer shifts to the blue spectral region. The opposite happens when the thickness of the layer increases upon UV light irradiation. This process of color change is reversible and can be repeated many times (Figures 2c and S6) yielding moderate narrowing of the spectral modulation range, likely because of the partial expulsion of free liquid crystal fraction from the network.^[^ ^22^, ^23^ ^]^

The design of the hydrazone switch allows its operation without causing disruption of the liquid crystalline order as we have demonstrated previously (see also discussion in Figure S7).^[^ ^19^ ^]^ Hence, the large molecular shape change upon photoisomerization is responsible for the building up of mechanical strain in the network, which causes its contraction/expansion. Since the manipulation of the color is a photochemically driven process, and the *E*/*Z* isomer ratio can be manipulated as a function of wavelength or irradiation time photostationary state, any color in the spectral modulation range can be obtained, and importantly, they can be locked in thanks to the extraordinary stability of the *E*‐hydrazone. For example, even after storing (in the dark) a sample pre‐exposed to blue light for as long as a year, no change in reflected color was observed, and the color was still changeable by UV exposure.

One can argue that similar effects can be achieved by using conventional azobenzene switches. To address this question, we prepared similar cholesteric coatings containing fluoro‐substituted azobenzene with very stable *Z*‐form.^[^ ^24^ ^]^ Our results (see Supporting Information, Figures S8 and S3) show that the azobenzene switch only allows a transmission band shift of 14 nm, which can be explained by the smaller molecular shape change (0.35 nm vs. 0.59 for hydrazone) and reduction of the liquid crystalline order by *Z*‐azobenzene.^[^ ^25^ ^]^ The latter effect usually causes expansion of the networks,^[^ ^26^ ^]^ which interferes with the contraction driven by the molecular shape change. On the other hand, the hydrazone switch designed by us does not affect the liquid crystalline ordering, and therefore, it generates a larger molecular change, resulting in around 5‐fold difference in color shift. This observation attests to the benefit of the hydrazone switch, which combines large geometrical changes and bistability with order‐to‐order transitions in bulk materials.^[^ ^27^, ^28^, ^29^, ^30^, ^31^, ^32^, ^33^, ^34^ ^]^

To prove that *Z*/*E* isomerization of the hydrazone indeed results in contraction of the network and reduction of its thickness, we studied the affected surfaces with AFM. We used a photomask with transparent and nontransparent stripes to impart topography on the cell surface topography using blue light exposure. Such patterning results in the alternation of exposed areas, where the network contracts and unexposed areas with native state of the network (Figure 3a). Figure 3b demonstrates the obtained color pattern observed by polarized light microscopy in reflection mode (see also Figure S9). The surface study of the stripe‐patterned coating performed by AFM revealed a thickness modulation as high as 75 nm (Figure 3c). From Equation (2), the expected modulation of the thickness is about 200 nm, considering an initial thickness of the coating of 2 µm and an average refractive index of 1.5. The lower experimental value can be explained by the partial erasure of the pattern because of the back *E*/*Z* isomerization of the hydrazone induced by the exposure to ambient (room) light during the experiments.

**Figure 3:**
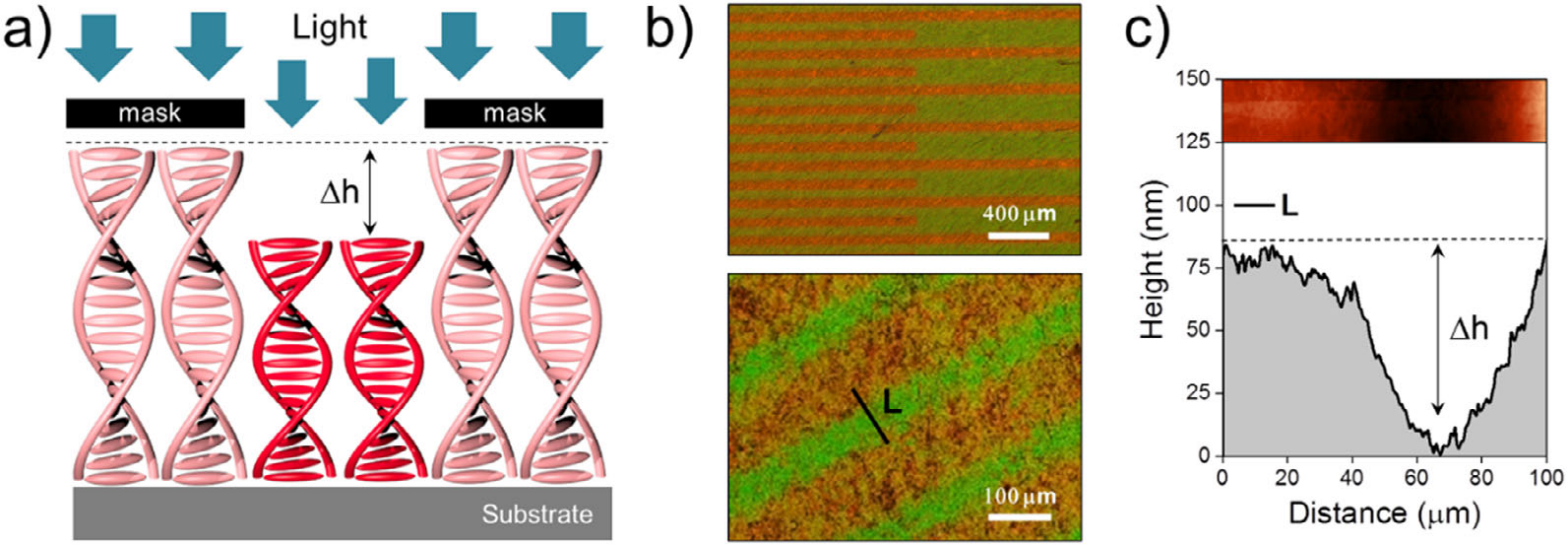
a) Recording of optical patterns on hydrazone‐based dynamic cholesteric networks. Irradiation of the cholesteric layer through a mask results in spatial modulation of the thickness and consequently color. b) Optical image of the striped pattern and the AFM surface profile c) along the track “L”, which displays the thickness drop in areas exposed to blue light. The corresponding AFM scan (25 µm width) is shown on top of Figure c.

In conclusion, we have demonstrated a supramolecular mechanochemistry approach to tunable optical coatings, where molecular shape changes in a hydrazone photoswitch translate into the mechanical contraction of a network, and further into structural color changes. The contraction of the cholesteric polymer network results from the tension applied by the hydrazone switch, which alters the pitch without disrupting the liquid crystal order. Unlike azobenzene‐based systems, which operate by a combination of both tension and order disruption, our approach to optical response preserves the ordering of the photonic scaffold while enabling reversible and durable color switching. The stability of both *Z* and *E* forms of the hydrazone ensures long‐term bistability, allowing for multiple cycles of actuation. We envision that this strategy—harnessing molecular‐scale mechanical inputs to control optical properties—can inspire new classes of responsive photonic coatings and tunable optical materials.

## Conflict of Interests

The authors declare no conflict of interest.

## Supporting information



Supporting Information

## Data Availability

The data that support the findings of this study are available from the corresponding author upon reasonable request.
