# Supplementary material for: Photo‐Controlled Dynamics of Cholesteric Polymer Coatings via Hydrazone Crosslinking
Source: Angew Chem Int Ed Engl. 2025 Jun 5;64(31):e202507358. doi: 10.1002/anie.202507358 (PMC12304863; doi:10.1002/anie.202507358)
Supplement: Supplementary file 1 — Supporting Information [file ANIE-64-e202507358-s001.pdf]

Supporting Information
©Wiley-VCH 2021
69451 Weinheim, Germany

Photo-Controlled Dynamics of Cholesteric Polymer Coatings via Hydrazone Crosslinking

Alexander Ryabchun, Yunita Florida, Quan Li, Rémi Plamont, Nathalie Katsonis, Ivan Aprahamian**

Abstract:. Developing responsive coatings and smart materials requires discovering a breadth of mechanisms by which external stimuli can be converted into useful signals. Here, we demonstrate an approach driven by supramolecular mechanochemistry, where mechanical input—molecular shape change—is translated into structural color variation. By embedding bistable, negatively photochromic hydrazone photoswitches into cholesteric polymer networks, we achieve a reversible, stable color shift through molecular-scale pulling and pushing of the photonic scaffold. Unlike azobenzene-based systems, which typically disrupt liquid crystal order, this approach modifies the pitch of a crosslinked cholesteric helix without loss of structural integrity. The long-lived bistability of both hydrazone isomers ensures durable optical switching. This concept provides a new strategy for designing mechanoresponsive photonic coatings and tunable optical materials.

DOI: 10.1002/anie.2021XXXXX

Table of Contents

Experimental Procedures 3
Materials 3
Cholesteric networks preparation 3
Measurements 3
Results and Discussion 3
Photostationary state study of hydrazone switch 3
Selective light reflection measurements 6
References 9

Experimental Procedures

Materials

The hydrazone-based photoswitch was synthesized following a previously reported procedure.[1] The fluorinated azobenzene diacrylate was synthesized following a previously reported procedure.[2] Liquid crystal diacrylate C6M, monoacrylate C6BP and chiral dopant CB15 were purchased from Synthon Chemicals and were used as received. Low molar mass liquid crystal mixture ZLI-1083 was composed from PCH3, PCH5 and PCH7 (all from Synthon) mixed in 30/30/40 proportion by weight. Photoinitiator Irgacure 819 was purchased from Sigma-Aldrich.

Cholesteric networks preparation

To promote adhesion of the polymer networks to the substrate, it was silanized with 3-(trimethoxysilyl)propyl methacrylate (Sigma-Aldrich) while another glass substrate was treated with perfluorodecyltriethoxysilane (Sigma-Aldrich) as described previously.[3]

The cholesteric photopolymerizable mixtures were prepared by mixing proper amounts (see Figure S3a) of crosslinkers, polymerizable LC monomers, low molar mass liquid crystal, chiral dopant and photoinitiator in dichloromethane follow by solvent evaporation and drying. Isotropization temperature of monomeric mixtures were in the range 29-31 °C. The monomeric mixture was introduced into the glass sandwich-like cell (with salinized substrate) followed by applying shear forces to promote planar alignment. A typical cell thickness of 2 µm was controlled by using a spacer (glass spheres). Photopolymerization was carried out at room temperature by exposing the cell to blue light (λ = 470 nm, intensity 43.7 mW/cm^2) for 90 seconds followed by post-curing at 50 °C for 5 minutes. Then the cell was opened with a razor blade yielding cholesteric layer supported on 3-(trimethoxysilyl)propyl methacrylate treated grass substrate.

Measurements

Absorbance spectra were recorded with UV/Vis spectrometer HR2000+ (Ocean Optics). Polarized light absorbance spectra were measured using spectrometer equipped with a polarizer (Glan-Taylor prism) mounted on a rotatable stage. Optical images were taken by means of polarized optical microscopy (Olympus BX51) in crossed polarizes and in reflection mode. The phase behavior was studied with a polarized optical microscope equipped with a heating stage (Instec). Surface topography was characterized by Atomic force microscopy (AFM) using an NTEGRA Spectra setup (NT-MDT) integrated with inverted microscope (Carl Zeiss). The images were recorded in tapping mode. Light emitting diodes (Thorlabs) with wavelengths of λ = 470 nm (I ≈ 43.7 mW/cm^2), λ = 340 nm (I ≈ 1.3 mW/cm^2), λ = 420 nm (I ≈ 338.4 mW/cm^2) were used for photopolymerization and photooptical studies. Intensity of light was measured with PM-100D power meter (Thorlabs).

Results and Discussion

Photostationary state study of hydrazone switch

Master solution containing 2% w/v solution of the hydrazone in chloroform was prepared in a small vial. About 0.5 mL of the master solution was transferred to a spectroscopic quartz cuvette. The solution was then irradiated at various wavelengths: 340 nm, 365 nm, 405 nm, and 420 nm for 30 minutes upon constant stirring. The resulting solution was then transferred to an NMR tube. The spectra of the photostationary states (PSSs) at each wavelength were recorded at ambient temperature on a Bruker Ascend™ 400 spectrometer operating at 400 MHz. The results of the initial form and irradiated solutions were then compared to calculate the *E*/*Z* ratio of each PSS. The photoisomerization of the hydrazone was examined by by UV/VIS spectroscopy as well (Figure S1).

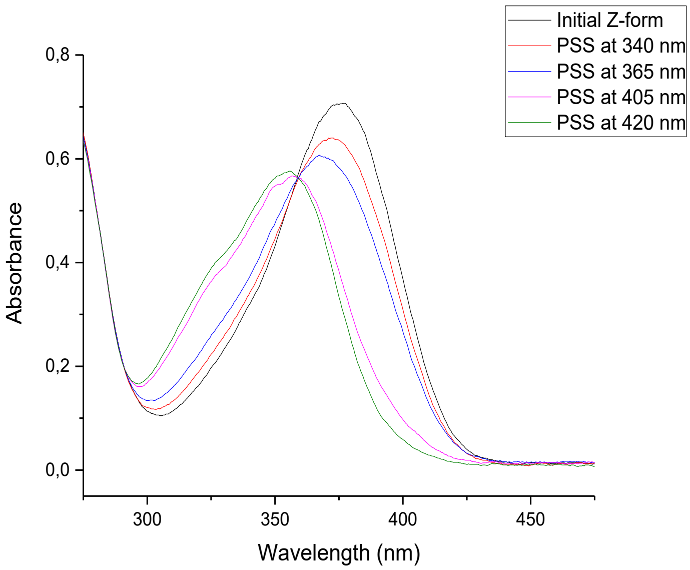

Figure S1. UV/Vis spectra of the hydrazone in chloroform upon irradiation at different wavelengths.

The *Z* form of hydrazone switch has a characteristic of a singlet peak of NH proton resonance at 14 ppm (Figure S2), which shifts to 11.5 ppm upon irradiation with visible light. The *Z/E* isomer ratio at different PSSs was calculated by comparing the integration of these two peaks using the following equations:.

$$\% \, Z - Hydrazone = \frac{I_{14}}{I_{14} + I_{11.5}} \times 100\%$$

$$\% \, E - Hydrazone = \frac{I_{11.5}}{I_{14} + I_{11.5}} \times 100\%$$

The obtained results are given in Figure S2 and go in line with UV-vis spectral measurements. The optimum irradiation wavelengths, λ = 420 nm and λ = 340 nm, yielding PSS of 94% of *E*-isomer and 83% of *Z*-isomers, were chosen for further studies of the cholesteric networks.

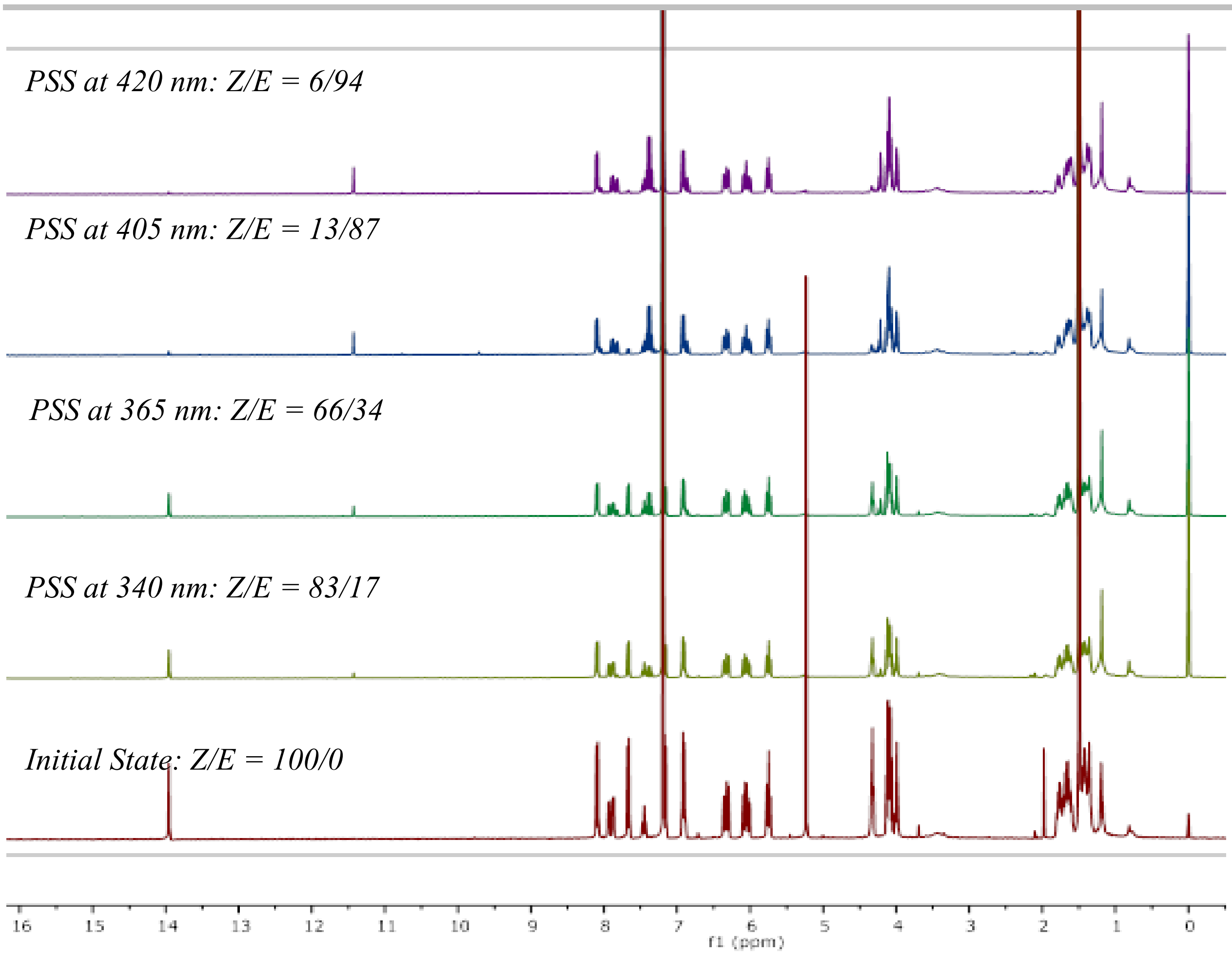

Figure S2. ^{1}H NMR spectra of the hydrazone ($CDCl_3$, RT) after irradiation at different wavelengths.

Selective light reflection measurements

a)

Compound	Mix 1	Mix 2	Mix 3	Mix 4	Mix 5	Mix 6**
HD	3	3	3	3	3	0
f-Azo	0	0	0	0	0	3
C6M	5	2	12	5	5	5
C6BP	0	0	0	10	22	0
CB15	30	30	30	30	30	30
ZLI-1083	62	65	55	52	40	62
T_{iso} (°C)	30.8	28.8	35	31.7	24.5	46.5

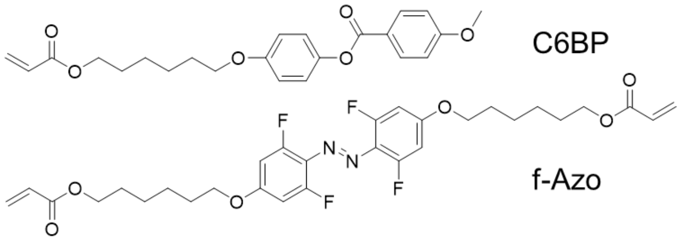

b)

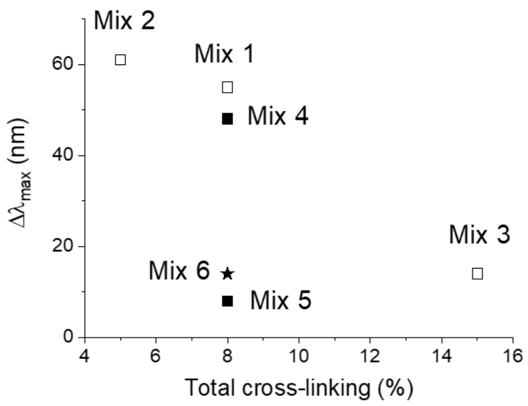

Figure S3. The range of selective light reflection tuning as a function of total cross-linking density of the cholesteric network. Concentration of hydrazone cross-linker and chiral dopant CB15 were kept constant at 3 and 30 wt%, respectively. Content of diacrylate C6M and low molar mass ZLI1083 were varied correspondingly. One network with additional 10 wt% of monoacrylate C6BP was tested as well (filled square).

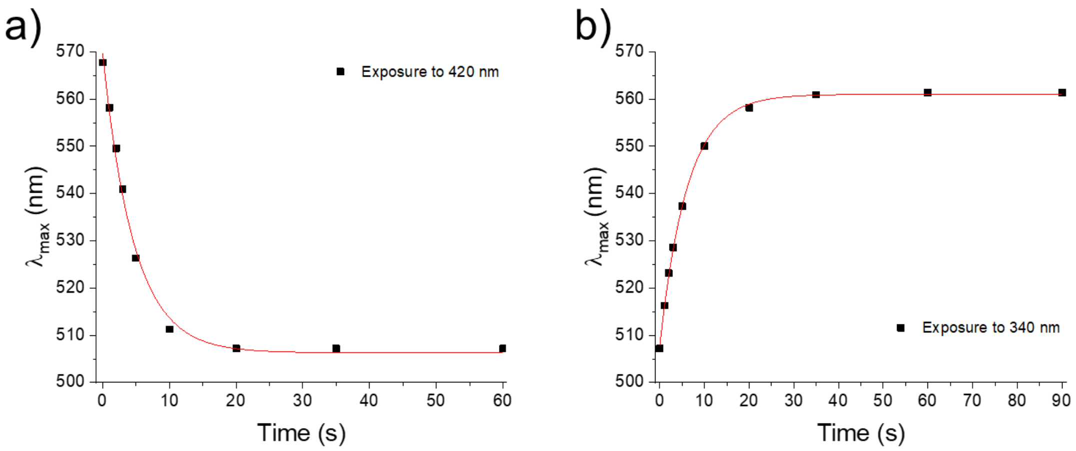

Figure S4. Dynamic of selective light reflection band shift upon exposure to (a) blue and (b)UV light.

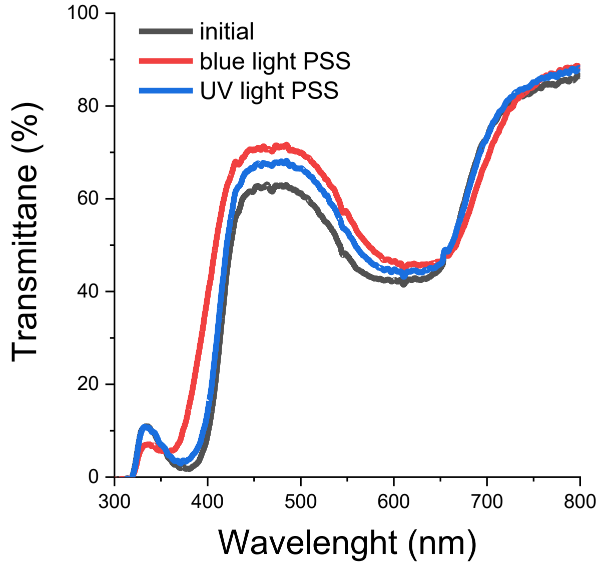

Figure S5. Transmission spectra of the cholesteric layer (Mix 1) prepared in the glass cell (between two glass substrates). Exposure with blue and UV light causes negligible bathochromic shift of the transmission band in contrast to lager hypsochromic shift when the layer is supported on a glass substrate (Figure 2b, main text). It is noteworthy that upon cell opening the networks are allowed to relax, and the volume reduction (approx. 4%) induced by the crosslinking results in the shrinkage of the network, thus causing the hypsochromic shift of transmittance band. [4]

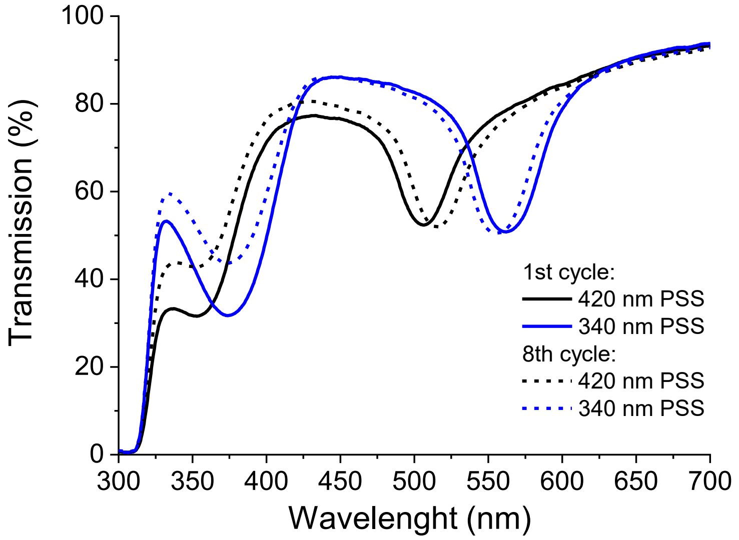

Figure S6. Transmission spectra of the cholesteric network after 1st and 8th cycle of blue/UV light exposure.

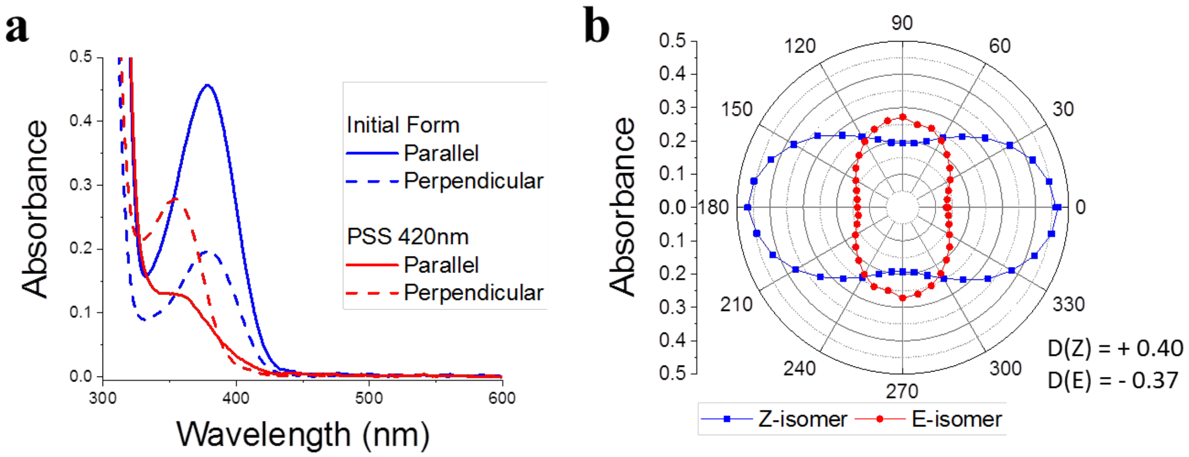

Figure S7. Molecular alignment of the hydrazone in the liquid crystal. Image (a) shows the polarized absorbance spectra of the hydrazone embedded into unidirectional aligned ZLI1083. Polar plot (b) shows the angular dependence of the polarized absorbance plotted at absorbance maximum. Values of dichroism (D) are provided in the figure.[1] The similar dichroic ratios (D values) observed for the E and Z isomers suggest that photoisomerization does not substantially alter the mesogen alignment, consistent with prior findings in systems where the hydrazone concentration was 1 wt% (Ryabchun et al., *J. Am. Chem. Soc.* **2019**, *141*, 1196).

Dichroism was estimated using the equation bellow to probe the ordering of the nematic host.

$$D = \frac{A_{\parallel} - A_{\perp}}{A_{\parallel} + A_{\perp}}$$

where $A_{\parallel}$ and $A_{\perp}$ denote the absorbance of polarized light, parallel and perpendicular to the liquid crystal alignment, respectively. D of 0 indicates no order, whereas 1 corresponds to a completely ordered alignment.

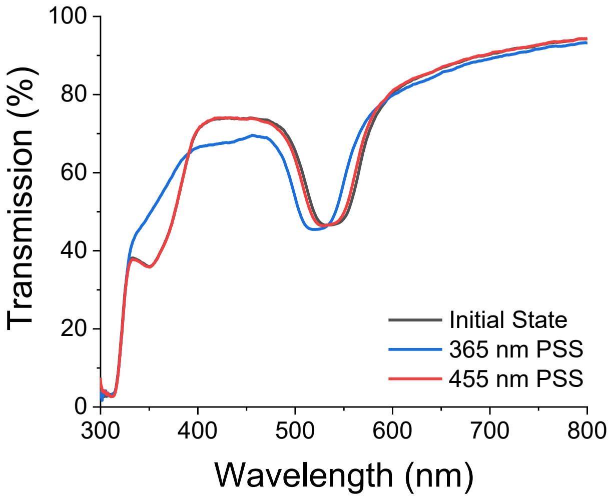

Figure S8. Transmission spectra of the cholesteric network containing fluorinated azobenzene based cross-linker (chemical structure is in Figures S3) obtained before irradiation, after irradiation with UV (365 nm) and after blue (455 nm) light. The shift of the reflection band is usually about 10-14 nm. PSS_{365} corresponds to *Z*-rich state (96% of *Z*-azobenzene) while PSS_{45} corresponds *E*-rich state (62% of E-azobenzene). Chemical composition of the network is similar to the one containing hydrazone with total cross-linking density of 8%.

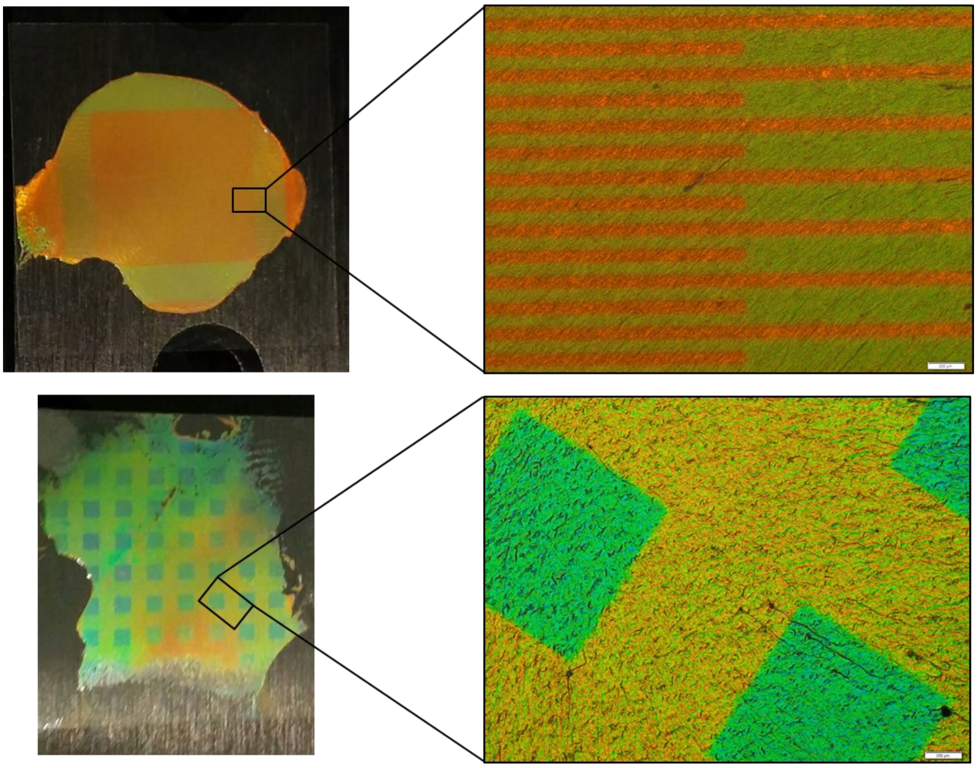

Figure S9. Examples of optical recording on hydrazone-based cholesteric networks obtained by mask exposure.

References

[1] A. Ryabchun, Q. Li, F. Lancia, I. Aprahamian, N. Katsonis, *J. Am. Chem. Soc.* **2019**, *141*, 1196.
[2] S. Iamsaard, E. Anger, S. J. Aßhoff, A. Depauw, S. P. Fletcher, N. Katsonis, *Angew. Chem. Int. Ed.* **2016**, *55*, 9908.
[3] J. E. Stumpel, E. R. Gil, A. B. Spoelstra, C. W. Bastiaansen, D. J. Broer, A. P. Schenning, A.P. *Adv. Funct. Mater.* **2015**, *25*, 3314.
[4] A. Ryabchun, I. Raguzin, J. Stumpe, V. Shibaev, A. Bobrovsky, *ACS Appl. Mater.Interfaces* **2016**, *8*, 27227
